# Differential Bacteriostatic Effects of Sucralose on Various Species of Environmental Bacteria

**DOI:** 10.1155/2013/415070

**Published:** 2013-09-30

**Authors:** Arthur Omran, Ronald Baker, Charles Coughlin

**Affiliations:** ^1^Department of Biology, University of North Florida, Jacksonville, FL 32224, USA; ^2^Florida Department of Health Bureau of Public Health Laboratories Jacksonville, FL 32202, USA

## Abstract

Sucralose was developed as a low-cost artificial sweetener that is nonmetabolizable and can withstand changes in pH and temperature. It is not degraded by the wastewater treatment process and thus has been found in waste water, estuaries, rivers and the Gulf Stream. Since the molecule can withstand heat, acidification, and microbial degradation, it is accumulating in the environment. The highest concentration of environmental sucralose detected to date is 300 ng/L. Our lab has isolated six bacterial species from areas that have been exposed to sucralose. We then cultured these isolates in the presence of sucralose looking for potential sucralose metabolism or growth acceleration. Instead we found something very interesting, bacteriostatic effects exhibited on all six isolates. This inhibition was directly proportional to the concentration of sucralose exposure. The efficiency of the growth inhibition seemed to be species specific, with various concentrations inhibiting each organism differently.

## 1. Introduction 

 An unexpected contaminant in our aquatic and costal environments is artificial sweeteners [1]. Due to the human inability to metabolize them, they are passed on to the environment via human excrement. Naturally the highest concentration of artificial sweetener contaminants is in waste water treatment plants' reservoirs. Artificial sweeteners such as saccharin and cyclamates are detected in lower concentrations and are found 90% degraded by the wastewater treatment process. Sucralose, however, is found in higher concentrations and is minimally degraded [2]. Degradation only occurs to a limited extent during hydrolysis, ozonation, and microbial processes indicating that breakdown of sucralose will likely be slow and incomplete leading to accumulation of sucralose in surface waters [3]. This indicates that conventional waste water treatment is ineffective at degrading sucralose [2]. From wastewater facilities the pollutants are dumped into public waterways, and sucralose has been detected in rivers in North Carolina, in the Gulf Stream, and even in the waters of the Florida Keys [4]. Also for the first time scientists are detecting sucralose in the USA inland surface waters [2]. Artificial sweeteners have been considered contaminants by environmental scientists for a short time because artificial sweeteners are water contaminants that are highly specific to wastewater [1].

Most artificial sweeteners are either partially or completely broken down due to the waste water treatment process using high temperatures, changes in pH, and constant filtration. It would seem that the exception is sucralose due to its ability to withstand drastic pH and temperature changes; it is also small enough to pass through the filtration process associated with the treatment. Hence, sucralose is being continuously released into our environment in ever increasing concentrations, and due to the human consumers' inability to metabolize this artificial sweetener, we are dumping it into our water ways and it is collecting over time [4]. As time passes sucralose will spread to other aquatic and costal ecosystems, increasing concentration [3]. The persistent qualities of sucralose may lead to chronic low-dose exposure with largely unknown consequences for human and environmental health [3]. It is also unknown what this increasing concentration of sucralose is doing to environmental microbes.

Sucralose may be acting on microbes by inhibiting growth. Studies of human oral and gut bacteria have shown an inhibition of bacterial growth in the presence of sucralose [5]. The same may be true for environmental microbes. In one study the incorporation of 126 mmol/L sucralose into glucose agar medium caused total inhibition of growth of *Streptococcus sobrinus* 6715-17, *Streptococcus sanguis* 10904, *Streptococcus sanguis* Challis, *Streptococcus salivarius*, and *Actinomyces viscosus* WVU627 [5]. In a related study rats were infected with *Streptococcus sobrinus* and given sucrose water diet, and they developed caries lesions. Then another group of rats was given the same bacteria but sucralose water instead of sugar water. Those rats had a drastic decrease in caries lesions in their teeth, demonstrating that oral bacteria cannot grow on the artificial sweetener hence causing less damage, proving that sucralose is noncariogenic [6]. These are good examples of sucralose inhibiting bacterial growth; however, there have been minimal studies on the inhibitory effects of sucralose on environmental microbes. 

Sucralose is increasing in concentration in our waterways, and it has been shown in previous studies to be harmful to oral and gut bacteria. We propose that sucralose can negatively affect environmental bacteria.

## 2. Methods and Materials

### 2.1. Collection of Bacteria

Water samples and soil samples from various test sites around Jacksonville Florida (Table 1) were collected aseptically using autoclaved collection flasks and jars. The samples were then used for microbial isolation.

### 2.2. Isolation of Bacteria

Fluid and soil extracted from samples were serially diluted with sterile 0.89% NaCl solution, then spread plated on Tryptic Soy Agar (TSA) (Difco Laboratories, Michigan, USA) plates infused with 80 mM sucralose, and incubated at 32.7°C for 48 h. Bacteria were isolated into pure cultures on subsequent TSA slants based on colony morphology (Table 2). Isolates of each bacterium were incubated (32.7°C) for one day for microbial analysis.

### 2.3. Microbial Analysis

 Individual isolates were extracted from pure culture and gram stained. The isolates were then analyzed using basic light microscopy to identify individual gram characters and cellular morphology (Table 2). Once a list of the isolates with their microbial characters was formed, they were screened for sucralose metabolism.

### 2.4. Sucralose Metabolism Inspection

In order to inspect organismal growth in the presence of sucralose, 0.1 mL of isolated cell cultures was diluted with 2.9 mL of 0.89% NaCl solution. These samples were streak plated onto M9 agar containing glucose (Technova, Nova Scotia, Canada) (positive control), M9 agar containing sucrose (positive control), M9 agar containing sucralose and glucose (experimental), M9 agar containing sucralose (experimental), and M9 agar containing no sugars (negative control). Isolates which exhibited growth on the M9 agar containing sucralose and glucose were selected for further experimentation (Table 3). This is due to the possibility that they may be resistant to or metabolizing sucralose, which was inspected during the growth testing experiment. The selected isolates were then identified via 16S rRNA sequencing.

### 2.5. Genomic DNA Extraction and PCR of 16S rRNA Gene

Genomic DNA was extracted from each of the selected bacterial isolates using the Ultraclean Microbial DNA Isolation Kit in accordance with the manufacturer protocols (MO BIO Laboratories, California, USA). The 16S rRNA gene was amplified using the bacterial consensus primers 8F (5′ AGTTGATCCTGGCTCAG 3′) and 1492R (5′ ACCTTGTTACGACTT 3′). The long polymerase chain reactions (PCR) consisted of 41.7 *μ*L dH_2_O, 5.0 *μ*L 10x *Taq* buffer, 1.5 *μ*L 50 mM MgCl_2_, 0.5 *μ*L 10 *μ*M forward primer 10 *μ*M reverse primer 0.4 *μ*L 25 mM dNTPs, 0.4 *μ*L 5 U/*μ*L *Taq* polymerase, and 1 *μ*L genomic DNA in a final volume of 50 *μ*L. DNA amplification was performed with the following thermocycler regime: 2 min at 98°C followed by 33 cycles of, 98°C for 30 s, 45°C for 60 s, 72°C for 90 s and a single step at 72°C for 10 min. Short PCR amplification consisted of 50 *μ*L reactions with analogous reagents/concentrations to the long PCR, using the additional primers 760R (5′ CTACCAGGGTATCTAAT 3′) and 790F (5′ ATTAGATACCCTGGTAG 3′) with the following thermocycler settings: 25 cycles of 98°C for 30 s, 44°C for 45 s, and 72°C for 90 s. 

### 2.6. PCR Cleanup, Cycle Sequencing, and Ethanol Precipitation

The short PCR products were cleaned up using the QIAquick PCR Purification Kit following the manufacturer protocols (Qiagen, California, USA). The four primers aforementioned were employed for cycle sequencing on a CEQ 8000 Genetic Analysis System (Beckman Coulter, California, USA) using 1 *μ*L GenomeLab DTCS Quick Start Master Mix, 2 *μ*L primer, 2 *μ*L DNA, and 7 *μ*L dH_2_O. Cycle sequencing consisted of 33 cycles at 96°C for 30 s, 37–47°C for 15 s, and 60°C for 4 min. Sequencing reactions were performed using each of the amplification primers and internal primers so that each fragment was sequenced in both the forward and reverse directions. Products were cleaned and precipitated according to the manufacturer specifications (Beckman Coulter, California, USA). 

### 2.7. Identification of Bacterial Species

The obtained sequences were compared to other sequences using the BLAST function through the NCBI website (http://www.ncbi.nlm.nih.gov/BLAST/). Sequences were determined 99% certain that the isolates were not new species. Isolates were then identified to the level of species (Table 2). 

### 2.8. Growth/Turbidity Testing

**Figure 1:**
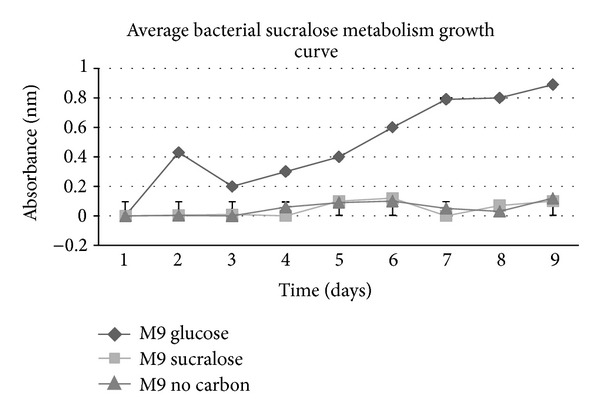
Composite growth curves for bacteria cultured in M9 media with various carbon sources.

Individual isolates were then cultured in Tryptic Soy Broth (TSB) media (Difco Laboratories, Michigan, USA) and incubated at 25°F. The control group consisted of 5 mL of TSB, and the experimental groups included 5 mL TSB with 0.5 mL of 10%, 20%, 30%, and 40% sucralose added, respectively. Turbidity of the cultures was measured over the next 9 days at the same time each day using a Sequoia Turner Ultraviolet Light Spectrophotometer set to 620 nm wavelength (Figure 1).

### 2.9. Sucralose Metabolism Validation

**Figure 2:**
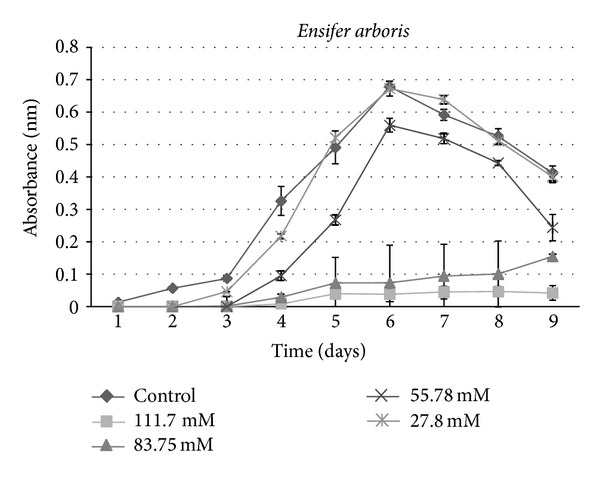
Growth curves for bacterial isolate: *Ensifer arboris *under varying concentrations of sucralose.

Individual isolates were cultured in M9 Broth media (Technova, Nova Scotia, Canada) and incubated at 25°C. The control group consisted of 5 mL of M9 broth with no carbon source; the experimental group included a 5 mL of M9 broth with sucralose as the only carbon source. Turbidity of the cultures was measured over the next 9 days at the same time each day using a Sequoia Turner Ultraviolet light Spectrophotometer set to 620 nm wavelength (Figure 2).

### 2.10. Disk Diffusion Assay and Determination of the Type of Inhibition

 Each bacterial isolate was spread plated into a lawn of confluent growth onto TSA media. Filter disks were prepared by whole punching out Whatman Grade Number 2V filter paper and impregnating the disks with 1.6 M sucralose. The antibiotic sensor disk was placed onto the surface of the media, 3 disks per petri dish. These samples were incubated overnight at 25°C. Diameters of the zones of inhibition were measured and recorded. The zones of inhibition were then swabbed and used to inoculate new TSA media. These re-culture plates were incubated over night at 25°C. The re-culture plates were then inspected for any growth. 

## 3. Results and Discussion

The glucose/100 mM sucralose M9 agar completely inhibited growth for 22 of the 28 isolates (results not shown). The isolates that were chosen for gene sequencing and further experimentation all were able to withstand the M9 sucralose and glucose agar (Table 3). Of the 28 isolates extracted from environmental samples, only 6 had growth on the sucralose/glucose media. They were identified as the bacteria *Microbacterium* sp. U13, *Stenotrophomonas* sp. I_61,* Rhizobium borbori*, *Citrobacter murlinlae*, *Ensifer arboris*, and* Streptomyces badius.*

These 6 isolates had fewer CFUs on the sucralose/glucose media than they had on the positive control groups of sucrose and glucose and had no growth on the sucralose only M9 agar (Table 3). These organisms were not metabolizing sucralose (Table 3, Figure 1).

Six unique bacterial isolates were obtained, of which four were Gram− and 2 Gram+ (Table 2). The isolates were subcultured in triplicate in the presence of 27.8 mM, 55.78 mM, 83.75 mM, and 111.7 mM sucralose to elucidate effects of sucralose on bacterial growth, with controls consisting of isolates emended with an additional volume of growth medium.

**Figure 3:**
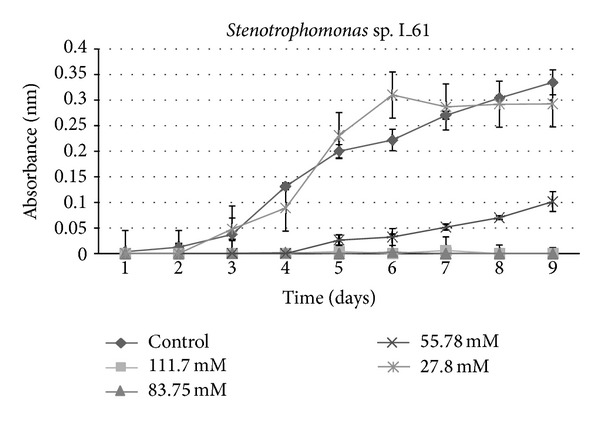
Growth curves for bacterial isolate: *Stenotrophomonas* sp. I_61 under varying concentrations of sucralose.

**Figure 4:**
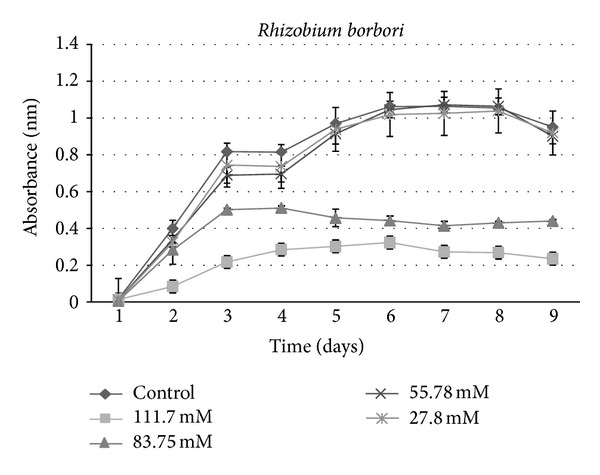
Growth curves for bacterial isolate: *Rhizobium borbori *under varying concentrations of sucralose.

**Figure 5:**
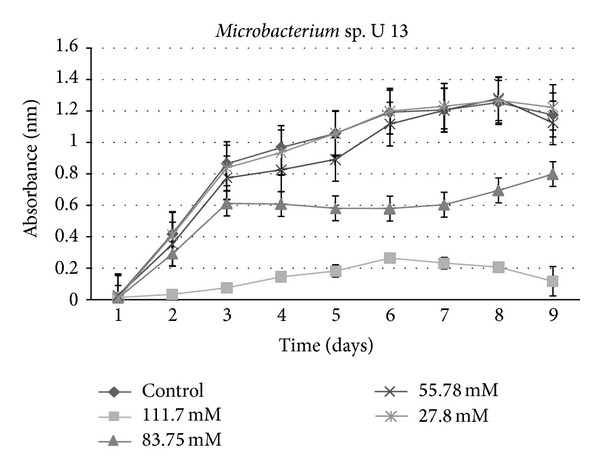
Growth curves for bacterial isolate: *Microbacterium *sp. U13 under varying concentrations of sucralose.

**Figure 6:**
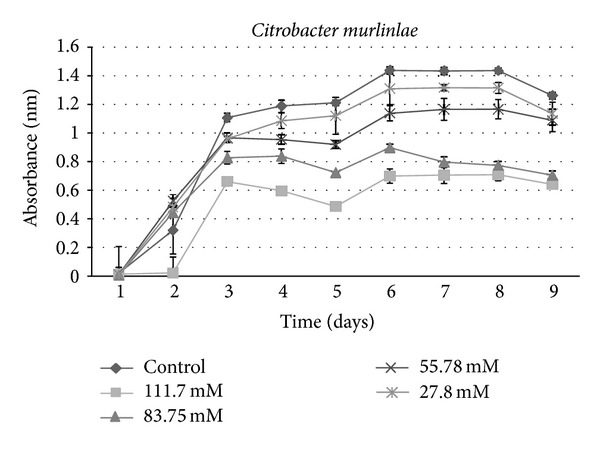
Growth curves for bacterial isolate: *Citrobacter murlinlae *under varying concentrations of sucralose.

**Figure 7:**
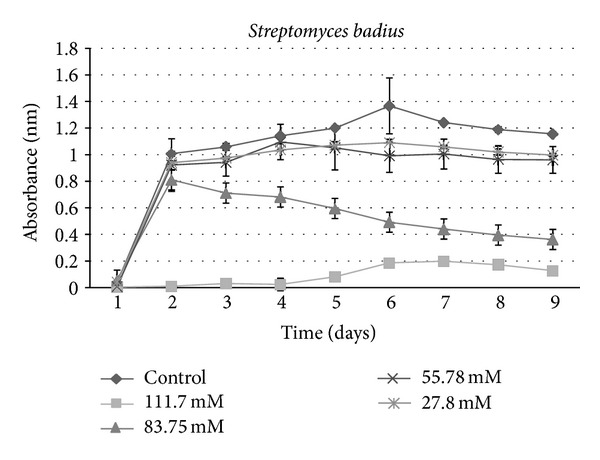
Growth curves for bacterial isolate: *Streptomyces badius *under varying concentrations of sucralose.

A growth curve showed a decrease in growth with those strains receiving sucralose addition compared to the control (Figure 1). An ANOVA indicated a significant (*P* < 0.001) difference between control groups and experimental strains amended with 83.75 mM and 111.7 mM sucralose, with experimental strains showing decreased growth rate (Figures 2, 3, 4, 5, 6, and 7). These results indicate that the addition of sucralose is a growth inhibitor for multiple strains of bacteria. 

We observed that there was no statistically significant (*P* > 0.99) difference between control groups and 25.7 mM for our isolates (Figures 2, 3, 4, 5, 6, and 7). The addition of low concentrations of sucralose (25.7 nm and less) had no significant effect on growth, whereas higher levels (83.75 mM and 111.7 mM) reduced growth drastically and possibly led to cell death. At 111.7 mM significant inhibition of total cell culture growth (Figures 3, 4, 6, and 7). Of those 6 bacterial isolates not completely inhibited by sucralose, two showed significantly decreased growth (*P* < 0.05) response in the presence of 55.78 mM sucralose (Figures 2 and 3). The negative effect that sucralose had on their growth rates was directly proportional to the concentration of sucralose added to the growth media. On average, the 27.8 mM and 55.78 mM sucralose treatments did not significantly inhibit the growth rates of these isolates or minimally inhibit the isolates in their growth rates. The 83.75 mM and 111.7 mM treatments did have a rather drastic inhibitory effect on bacterial growth across the board. The inhibition exhibited on all isolates was similar to that of previous studies, in which sucralose causes total growth inhibition of oral bacteria in lab mice [5, 7].

Disk diffusion assay data indicated differential growth inhibition as well. Regrowth from inhibited zones was tested; regrowth indicated a bacteriostatic inhibition for each bacterial isolate. This means that sucralose is a bacteriostatic agent (Table 4). This study is limited in its scope, as it is an inquiry of pure science. This is due to current environmental concentrations being in the 300 ng/L and below range. The research is meant to investigate the possibility of growth inhibition occurring in environmental isolates in pure culture. 

## 4. Conclusions

The current environmental concentrations of sucralose (300 ng/L in waste water and less in fresh waters) do not seem to have any effect on bacterial growth. Sucralose is, however, increasing in its concentration due to its inability to be degraded by pH and temperature changes [2] and its nonmetabolism by microbes (Figure 2). Sucralose would at higher concentrations, potentially 55.78 mM, hurt the bacterial community. This type of contamination would take a very long time to accumulate; however, it is troubling because the bacterial community is the basis for the health of many ecosystems. 

## Figures and Tables

**Table 1:** A list of environmental sample sites.

Sample number	Location	Sample type	GPS coordinate northwest
1	Lake Oneida	Water and soil	30.266912, −81.513347
2	The Rudder Club Dock (St. Johns River)	Water and soil	30.193071, −81.691266
3	Duval County Dock (St. Johns River)	Surface water	30.165346, −81.645559
4	St. Johns Parkway Dock (St. Johns River)	Surface water	30.045679, −81.667192
5	Clay County WasteWater Facility	Nutrient poor wastewater	30.093079, −81.764524
6	St. Johns County Waste Facility	Purified wastewater product	30.106153, −81.625693
7	Guana River Road (Estuary)	Water and soil	30 01′23.04–81 19′42.21

**Table 2:** Isolate identity and morphology.

Organism identity based on 16s rRNA gene	Gram character	Shape	Colony morphology
*Microbacterium * sp. U 13	Gram+	Cocci	Greyish pale, filamentous flat with filiform margins
*Stenotrophomonas * sp. I_61	Gram−	Cocci	Yellowish white, circular umbonate form with entire margins
*Rhizobium borbori *	Gram−	Cocci	Grey, circular convex form with entire margins
*Citrobacter murlinlae *	Gram−	Basili	Bright white, umbonate form with entire margins
*Ensifer arboris *	Gram−	Basili	Dull white, rhizoid form with filiform margins
*Streptomyces badius *	Gram+	Basili	Bright white, filamentous form with filiform margins

**Table 3:** Initial CFU counts for isolate selection.

Organism identity based on 16s rRNA gene	CFU count on M9 agar with glucose	Sucrose	Glucose and 80 mM sucralose	Sucralose	No sugars
*Microbacterium* sp. U 13	281	201	45	0	0
*Stenotrophomonas* sp. I_61	233	80	40	0	0
*Rhizobium borbori *	171	126	32	0	0
*Citrobacter murlinlae *	262	99	60	0	0
*Ensifer arboris *	285	140	63	0	0
*Streptomyces badius *	294	133	88	0	0

**Table 4:** Disk diffusion assay data, and zone of inhibitions are indicated. Regrowth from inhibited zones was tested; regrowth indicated a bacteriostatic inhibition not bactericidal.

Isolate	Regrowth	Diameters of inhibition (mm)						Average inhibition
*Microbacterium* sp. U 13	yes	1.12	1.1	1.5	1	1.05	1	1.12
*Stenotrophomonas* sp. I_61	yes	2	2.1	2.3	1.5	1.6	1.9	1.9
*Rhizobium borbori *	yes	1.1	0.8	1.1	1.15	1.3	1	1.07
*Citrobacter murlinlae *	yes	1.3	1	0.9	0.8	1	1	1
*Ensifer arboris *	yes	0.9	0.9	0.8	0.7	0.9	1	0.866
*Streptomyces badius *	yes	0.5	0.6	0.7	0.7	0.5	1	0.667
